# Should students still fear social media? Conveying an online personality can enrich the doctor–patient relationship

**DOI:** 10.3402/meo.v19.25752

**Published:** 2014-09-24

**Authors:** Nitesh V. Mehta, Mohith Shamdas, Thomas J. Arnold

**Affiliations:** Department of Medicine, Imperial College London, London, UK

As a revolutionary means of interacting with others, social media networks – and the personal information they contain – have presented a quandary for the modern practice of medicine. In a profession which defines itself so intently on the sanctity of the ‘doctor–patient’ relationship, the public sharing of personal information represents a radical shift in what is readily ‘knowable’ about whom.

Recently, Clyde and his associates found that subjects responded more favourably to ‘healthy’ personal profiles, such as those displaying hobbies or activities (e.g., hiking, reading), than to strictly professional profiles (1). This is understandable, since initial impressions are easily formed when viewing an online personal profile, and these more personal attributes reflect a more grounded, humanistic character desired in our doctors and teachers.

Arguably, the inclusion of personal interests and hobbies may promote richer ‘doctor–patient’ and ‘student–teacher’ relationships, both of which are essential to patient care, medical professionalism, and identity development. Of course, just as certain attributes, values, or behaviours can enhance professional relationships, others can be potentially damaging.

This proverbial ‘double-edged sword’ applies equally to medical students, whose actions now, given the permanent nature of online content, could one day exert unanticipated effects on future careers. For example, with the advent of LinkedIn, job recruiters can routinely scan social networks (2), making it prudent to assume that online personal profiles are just as easily accessible to potential employers as family and close friends. Thus, maintaining a ‘healthy’ online personal profile may not be simply professional: it may also enhance trainees’ future employability.

As medical students, we are repeatedly warned of the dangers of social networks and advised to restrict our profiles and carefully monitor our privacy settings. While being well-informed about what *not* to post online is of obvious importance, medical training too often emphasises the more negative aspects of social media, rather than encouraging its use in a positive, professional manner.

It is perhaps not surprising, then, that social media networks have become somewhat stigmatised in the medical profession; in their use, we are taught to be cautious and wary, rather than how to acceptably convey the well-roundedness that contributed to our selection as future doctors. In light of studies by Clyde and colleagues, it is clear that patients are increasingly aware of physicians’ Internet presence. Recognising this, students should be coached in the responsible use of such platforms to nurture the confidence of patients and colleagues, and not discouraged for fear of potential misuse or abuse. In this endeavour, patients stand to gain as much as those who will one day care for them.

## Conflict of interest and funding

All authors have completed the ICMJE uniform disclosure form at www.icmje.org/coi_disclosure.pdf and declare: no support from any organisation for the submitted work; no financial relationships with any organisations that might have an interest in the submitted work; no other relationships or activities that could appear to have influenced the submitted work.

*Nitesh V. Mehta* Department of Medicine Imperial College London London, UK Email: nitesh.mehta09@imperial.ac.uk *Mohith Shamdas* Department of Medicine Imperial College London London, UK *Thomas J. Arnold* Department of Medicine Imperial College London London, UK
